# Tumor-nerve crosstalk: peripheral mechanisms mediating bone tumor progression and cancer-induced bone pain

**DOI:** 10.3389/fonc.2026.1906776

**Published:** 2026-09-08

**Authors:** Huirong He, Lingduo Zhang, Yingying Zhang, Su Yi, Lihua Hang

**Affiliations:** 1 Gusu School, Nanjing Medical University, Kunshan First People’s Hospital, Kunshan, China; 2 Department of Anesthesiology, Kunshan Hospital Affiliated to Jiangsu University, Kunshan, China; 3 Kunshan Cancer Pain Prevention and Treatment Key Laboratory, Kunshan, China

**Keywords:** bone metastasis, cancer neuroscience, cancer-induced bone pain, mitochondrial transfer, neural remodeling, tumor microenvironment, tumor–nerve crosstalk

## Abstract

Cancer-induced bone pain (CIBP) is one of the most debilitating complications of primary bone tumors and bone metastases. Traditionally regarded as a consequence of bone destruction and mechanical compression, CIBP is now recognized as a complex pathological process involving dynamic interactions among tumor cells, peripheral nerves, immune cells, and the bone microenvironment. Increasing evidence indicates that tumor–nerve crosstalk contributes not only to nociceptive sensitization and neural remodeling but also to tumor progression and immune regulation. In the bone tumor microenvironment, tumor-derived mediators, including inflammatory cytokines, neurotrophic factors, and acidic metabolites, activate and sensitize intraosseous nociceptors, promoting peripheral sensitization and pathological nerve sprouting. Conversely, neural components can shape the tumor microenvironment through neurotransmitter signaling, immune modulation, and stromal remodeling and, in selected experimental models, influence tumor growth and metastatic adaptation. Recent advances in cancer neuroscience have further expanded this concept beyond classical biochemical communication. Emerging studies indicate that cancer cells can acquire neuron-derived mitochondria, with tunneling nanotube-like structures implicated in experimental coculture systems. Although this process may enhance tumor metabolic adaptation, its relevance to neural dysfunction and CIBP remains unclear. This review summarizes current evidence regarding the peripheral mechanisms of tumor–nerve interactions in CIBP, with particular emphasis on neural remodeling, neuro-immune communication, mitochondrial transfer, and emerging therapeutic targets. We distinguish established CIBP mechanisms from model-dependent observations and untested hypotheses and discuss the translational limitations of targeting the tumor–bone–nerve interface. This evidence-based distinction may facilitate the development of mechanism-based interventions that alleviate pain without overstating their anticipated effects on tumor progression.

## 1 Introduction

Statistics indicate that over 60% of patients with advanced-stage cancer experience clinically significant pain (1). Notably, beyond primary malignant bone tumors, the incidence of bone metastasis is high during the progression of breast, prostate, renal, and lung cancers; consequently, more than 80% of patients with metastatic bone disease may experience significant bone pain (2). This pain typically manifests as persistent background pain coupled with breakthrough episodic pain induced by mechanical stimuli (e.g., weight-bearing or ambulation), with major effects on physical function, psychological state, and overall quality of life (2, 3). Therefore, the alleviation and management of cancer-induced bone pain (CIBP) constitute an indispensable component of oncological care. Conventional clinical management relies on the WHO analgesic ladder, primarily utilizing non-steroidal anti-inflammatory drugs (NSAIDs) and opioids, together with disease-directed therapy, radiotherapy, and antiresorptive treatment (2–4). Nevertheless, inadequate analgesia and treatment-limiting adverse effects remain common (3, 4). These traditional approaches do not uniformly address the multiple tumor-, bone-, immune-, and nerve-derived drivers of CIBP.

In recent years, the understanding of cancer-induced bone pain (CIBP) has undergone a paradigm shift. It is no longer viewed merely as a byproduct of structural bone destruction or passive physical compression caused by tumor growth. Instead, CIBP is now recognized as the result of dynamic, bidirectional, and multidimensional interactions among tumor cells, the nervous system, immune cells, and the bone microenvironmental matrix (4–6). Tumor cells and associated cells within the bone microenvironment—including osteocytes, stromal cells, and immune cells—secrete an array of algogenic mediators (4, 6). These mediators activate and sensitize intraosseous nociceptors, representing a fundamental molecular mechanism of CIBP (4, 6). Tumor cells can also recruit and remodel peripheral nerve fibers (7–10). In non-bone cancer models, tumor cells may engage in direct organelle exchange with neurons, including nerve-to-cancer mitochondrial transfer; however, this mechanism has not been established in CIBP (11). Concurrently, sensory neurons can release neuropeptides such as CGRP and Substance P and thereby influence immune and stromal components of the tumor microenvironment, although these effects are cancer-type and model dependent (7, 12–14). At a broader systems level, chronic psychological stress may influence tumor progression through coordinated neuroendocrine, immune, inflammatory, and metabolic pathways. Persistent activation of the hypothalamic–pituitary–adrenal axis and sympathetic nervous system can alter glucocorticoid and catecholamine signaling, thereby reshaping immune surveillance, inflammatory responses, oxidative balance, and tumor metabolic adaptation (15). These systemic mechanisms provide broader context for cancer neuroscience, but they are distinct from the local peripheral tumor–bone–nerve interactions emphasized in this review, and their specific contribution to CIBP remains unclear.

Against this backdrop, the present review aims to systematically evaluate recent advances regarding the role of tumor–nerve crosstalk in the initiation, maintenance, and therapeutic management of CIBP. We distinguish mechanisms directly demonstrated in bone tumor pain models from findings derived from non-bone cancers, neuropathic pain, or *in vitro* systems, and identify the methodological and translational limitations that should guide future research.

## 2 Multi-level network regulation of the nervous system by tumors and CIBP

The regulatory effects of tumors on the nervous system are multifaceted. In primary bone tumors and bone metastases, tumors do not merely exert mechanical stress through physical compression; they also modulate neuronal structure and function by secreting bioactive factors (4–7). This biochemical signaling triggers neural remodeling, peripheral sensitization, and peripheral neuropathic alterations, which collectively facilitate the initiation and maintenance of CIBP. The relative contribution of each process varies with tumor type, lesion location, disease stage, and the balance between osteolytic and osteoblastic remodeling.

### 2.1 Innervation of bone and the pain “generator”

Peripheral nociceptive signals are conveyed through DRG neurons to the spinal cord, whereas subsequent spinal and supraspinal processing lies beyond the scope of this review.

Bone tissue is not an inert structure but is characterized by rich and specialized innervation. Research has demonstrated that intraosseous nerve fibers primarily consist of sensory and sympathetic nerves, which penetrate the bone via the periosteum, Haversian canals, and marrow vascular channels. Notably, the periosteum possesses the highest density of nerve fibers, serving as a critical peripheral origin of bone pain (16). The sensory component is predominantly composed of peptidergic nociceptive fibers (primarily peripheral Aδ and C fibers) expressing calcitonin gene-related peptide (CGRP), Substance P, or tropomyosin receptor kinase A (TrkA). These fibers are responsible for transducing stimuli into electrical signals and modulating bone remodeling and inflammatory responses through the release of neuropeptides. In contrast, sympathetic nerves typically express noradrenergic markers such as tyrosine hydroxylase (TH) and are involved in the regulation of bone blood flow, metabolism, and immune functions (4, 16–18). Recent studies further indicate that intraosseous nerves do not merely transmit pain signals; they also establish a sophisticated bidirectional communication network with tumor cells, immune cells, and stromal cells. This network plays a pivotal role in the progression of bone metastasis and cancer-induced bone pain (CIBP), suggesting that bone innervation serves the dual functions of sensory transduction and microenvironmental regulation (19) (**Table 1**).

**Table 1:** Major neural populations and nociceptor phenotypes relevant to CIBP.

Nerve fiber type	Key molecular markers	Physiological function	Role in cancer-induced bone pain (CIBP)
Peptidergic Sensory Nerves	CGRP, Substance P, TrkA	Mechanosensation, regulation of bone remodeling	Signal transduction, pathological sprouting, neurogenic inflammation
Sympathetic Nerves	Tyrosine Hydroxylase (TH), NPY	Vasomotor control, HSC niche regulation	Release of norepinephrine (NE) to promote tumor growth and metabolism
Non-peptidergic Nociceptors	P2X3, Ret (P2X3 evidence is strongest in DRG/peripheral nociceptors; direct intraosseous P2X3-positive fiber evidence remains limited)	Cutaneous nociception	ATP-sensitive nociceptive signaling; potential role in bone microenvironment remains under investigation rather than established as a major intraosseous fiber subtype.

### 2.2 Physical compression, invasion, and structural bone destruction

In the peripheral mechanisms of cancer-induced bone pain (CIBP), both direct and indirect interactions between the tumor and neural components contribute to pain generation. As the tumor expands within the bone, tumor-induced bone destruction and pathological remodeling may result in trabecular disruption, cortical thinning, microfractures, increased intraosseous pressure, and structural instability, thereby mechanically compressing, stretching, or directly injuring intraosseous and periosteal nerve fibers (4). These structural changes are particularly relevant to movement-evoked and breakthrough pain, because weight-bearing or local mechanical loading can further deform the weakened bone and periosteum. Nerve entrapment may subsequently cause localized ischemia and mechanical damage, whereas direct tumor invasion can disrupt the structural integrity and function of adjacent nerves. Furthermore, cancer cells may spread along or invade peripheral nerves through a specialized process known as perineural invasion (PNI), thereby causing additional neural injury (20). Thus, physical compression, tumor invasion, and loss of skeletal integrity are important contributors to the initiation and exacerbation of CIBP.

However, clinical observations indicate that the severity of pain does not exhibit a fixed linear relationship with the radiographic extent of bone destruction. Relatively small lesions may produce severe and refractory pain, whereas some radiographically extensive, predominantly osteoblastic lesions may remain asymptomatic or cause only modest pain. Therefore, structural bone alteration should be regarded as an important contributor to CIBP, but not as a simple surrogate measure of pain severity (4, 21). This clinicoradiological dissociation may be explained by differences in lesion location, local nerve density, periosteal involvement, cortical integrity, mechanical instability, microfracture formation, and intraosseous pressure. In addition, the pain-generating capacity of a lesion depends on its accompanying biological activity, including osteoclast-mediated acidification, the release of inflammatory and algogenic mediators, direct nerve injury, and pathological neural remodeling. Predominantly osteoblastic lesions may generate conspicuous radiographic sclerosis without producing an equivalent degree of cortical collapse, periosteal distortion, or osteoclast-associated extracellular acidification; nevertheless, these lesions can become painful when mixed osteolytic activity, microfractures, mechanical instability, or neural involvement is present (21, 22). Accordingly, the discordance between skeletal lesion burden and pain intensity further indicates that structural damage interacts with inflammatory, nociceptive, and neuropathic mechanisms rather than determining CIBP severity in isolation.

### 2.3 Chemical sensing and signal sensitization in the tumor microenvironment

Upon colonization of bone tissue, tumor cells drive a profound remodeling of the bone microenvironment, establishing a pathological niche conducive to their survival and expansion. By secreting an array of cytokines and growth factors—such as PTHrP, TGF-β, IL-1, and IL-6—and by altering RANKL/RANK/OPG signaling, tumor cells disrupt the homeostatic balance of bone remodeling (23–25). This disruption fosters osteoclast activation and impairs osteoblast function, leading to accelerated bone resorption and matrix degradation. Growth factors released during matrix breakdown can further stimulate tumor proliferation, forming a tumor–bone feedback loop (23, 24). Beyond osteoclast-driven resorption, osteoblast dysfunction and oxidative injury may also contribute to remodeling imbalance. In a titanium nanoparticle-induced periprosthetic osteolysis model, Zhong et al. found that TCF4-dependent transcription of YTHDF1 enhanced GPX4 and SLC7A11 translation, thereby suppressing osteoblast ferroptosis and alleviating osteolysis (26). Separately, Wang et al. demonstrated that binding of sclerostin loop3 to LRP4 facilitated the interaction of sclerostin with LRP6, thereby antagonizing osteoblastic Wnt/β-catenin signaling and inhibiting bone formation (27). Although neither study used a tumor model, these findings provide complementary osteoblast-centered mechanisms that may inform future investigation of tumor-associated bone destruction. Simultaneously, tumor metabolism results in localized acidification, hypoxia, and the accumulation of pro-inflammatory factors. These changes activate immune cells, stromal cells, and fibroblast-like cells, which collectively remodel the extracellular matrix (ECM) and exacerbate neural sensitization (7, 28–30).

Accumulating evidence indicates that intraosseous sensory and sympathetic nerves are integral to this process. Under tumor-associated conditions, nerve fibers undergo regeneration and phenotypic changes and communicate bidirectionally with tumor cells through neurotrophic factors, neuropeptides, and neurotransmitters (7, 31). This crosstalk can influence tumor progression and amplify nociceptive signaling. Recent work has also demonstrated nerve-to-cancer mitochondrial transfer in experimental breast cancer and metastatic models (11). This finding expands the possible modes of tumor–nerve communication, but it has not been demonstrated in bone tumor pain models.

Rather than constituting a single uniform “vicious cycle,” these interactions can be separated into partially overlapping feedback axes. The tumor–bone axis is supported by evidence that tumor-derived factors promote osteoclast-mediated bone resorption, which releases matrix-stored growth factors that further support tumor growth (23–25). The tumor–nociceptor axis involves algogenic mediators, extracellular acidosis, and neurotrophic signaling that activate, sensitize, or structurally remodel sensory fibers (6, 7, 10). A reciprocal nerve-to-tumor axis has been demonstrated in selected cancer models through neurotransmitter, neuropeptide, stromal, immune, and metabolic mechanisms (11, 14, 32), but its contribution to bone metastasis and CIBP is less consistently established (7, 32). These axes may coexist, but their relative importance varies with cancer type, lesion phenotype, disease stage, and experimental model (7, 21).

#### 2.3.1 Liberation of chemical factors from the tumor/bone microenvironment inducing neural sensitization and activation

**Figure 1:**
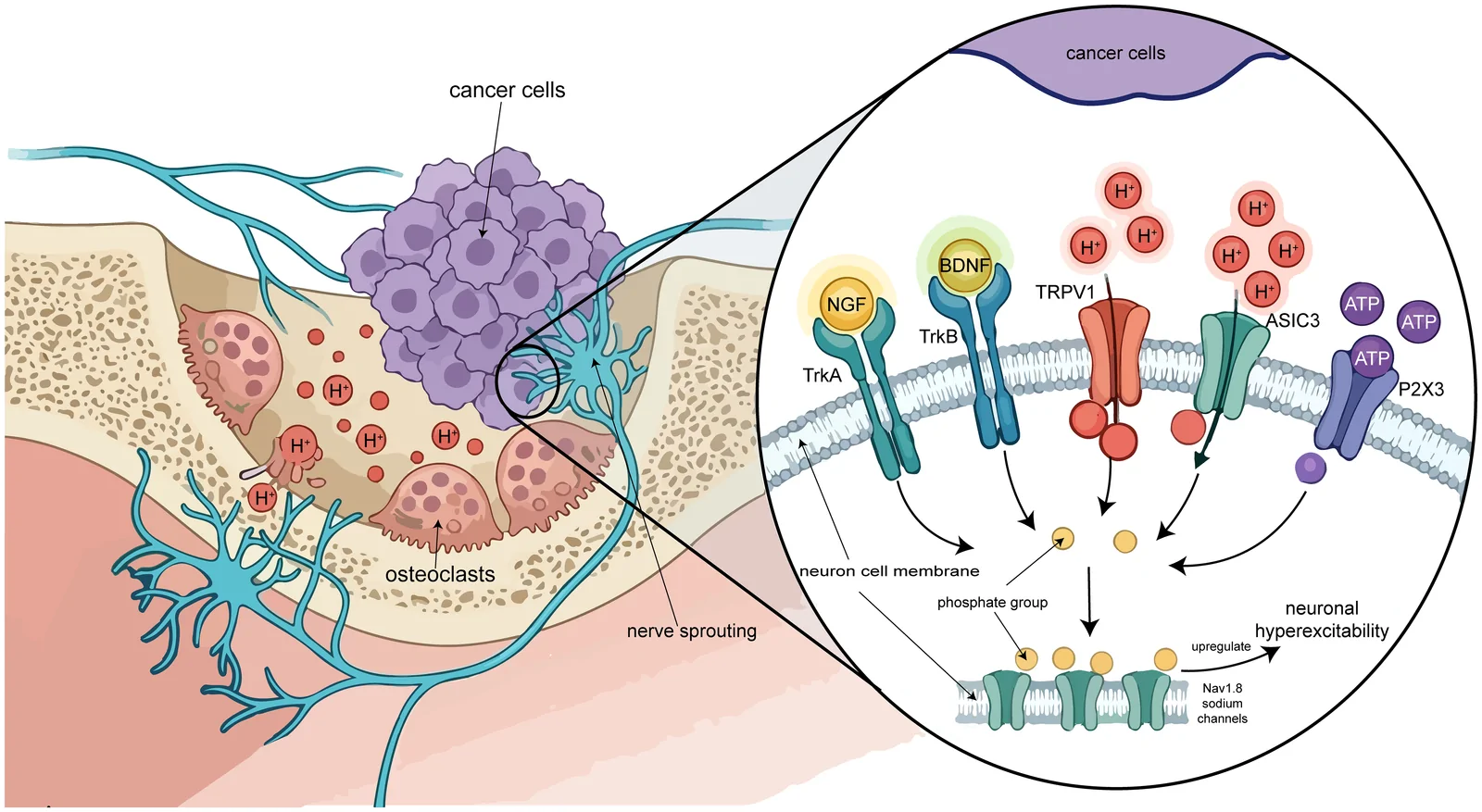
Peripheral sensitization and the “algogenic cocktail” in the bone tumor microenvironment.

Extensive research using various animal models of cancer-induced bone pain (CIBP), including murine and rat models of breast cancer, prostate cancer, osteosarcoma, and melanoma bone lesions, has demonstrated that tumor cells, together with osteoclasts, stromal cells, endothelial cells, macrophages, mast cells, and T cells, release a diverse array of algogenic mediators. These include protons (H^+^), pro-inflammatory cytokines (TNF-α, IL-1β, IL-6 and GM-CSF), nerve growth factor (NGF), brain-derived neurotrophic factor (BDNF), adenosine triphosphate (ATP), prostaglandin E2 (PGE2), endothelin-1 (ET-1), bradykinin, chemokines such as CCL2/CXCL12, and reactive oxygen species (5, 7, 33, 34). These mediators act on ion channels and receptors located on intraosseous and periosteal nerve endings, including ASIC3, TRPV1, P2X3, TrkA, TrkB, endothelin A receptors, EP receptors, cytokine receptors, and voltage-gated sodium channels such as Na_v_1.8/Na_v_1.9. Mechanistically, tumor acidosis gates TRPV1 and ASIC3; ATP activates P2X3/P2X2/3 receptors; NGF-TrkA signaling increases TRPV1 and Na_v_ channel expression through PI3K/MAPK pathways and retrograde transport to DRG neurons; PGE2-EP signaling activates cAMP/PKA-dependent phosphorylation of Na_v_ channels; and TNF-α/IL-1β/IL-6 enhance nociceptor excitability through NF-κB, JAK/STAT, p38 MAPK and ERK signaling. Together, these changes lower activation thresholds, increase ectopic or spontaneous firing, and enhance nociceptive responses to mechanical loading, thereby contributing to movement-evoked pain and episodic pain exacerbations (**Figure 1**). Thus, the phrase “amplifying pain signaling” refers not only to stronger peripheral input but also to receptor up-regulation, ion-channel phosphorylation, altered transcription in DRG neurons, and increased spontaneous or mechanically evoked firing (7). Evidence strength differs among targets: NGF-TrkA, TRPV1, and pathological sensory sprouting have direct support in bone cancer models (10, 35), whereas the bone-specific contribution of some receptors, including P2X3-positive intraosseous fibers, remains less firmly established (7) (**Table 2**).

**Table 2:** Molecular mediators and receptors involved in peripheral sensitization in cancer-induced bone pain, with species/model and cancer-context annotations.

Mediator/receptor	Ligand, species/model and cancer context	Mechanistic role in CIBP
TrkA	NGF; mouse/rat CIBP models; breast/prostate bone metastasis and osteosarcoma contexts	Retrograde signaling increases TRPV1/Na_v_ expression, promotes CGRP+/GAP43+ sprouting and neuroma-like structures, and sustains movement-evoked pain.
TRPV1	Acidic pH, heat and inflammatory lipids; rodent bone cancer pain models; osteolytic acidic tumor-bone microenvironment	Acidosis-sensitive nociceptor activation; contributes to mechanical allodynia and breakthrough pain.
ASIC3	Mild extracellular acidosis; rodent sensory neurons and CIBP models; acidified metastatic niche	Sustained inward currents and spontaneous firing during subtle pH changes.
P2X3/P2X2/3	ATP from necrotic or stressed tumor/immune cells; rodent DRG/peripheral nociceptor studies; direct intraosseous fiber evidence remains limited	ATP-gated cation influx enhances nociceptor excitability; bone-specific role should be presented as plausible/under investigation.
TrkB	BDNF; rat CIBP models and neuropathic pain studies	Supports peripheral and central sensitization and pain-aversion pathways.
ETA receptor	ET-1; rodent cancer pain models; tumor-cell expression in prostate, breast and other cancers	Direct excitation of nociceptors and contribution to intense ongoing pain.
EP receptors	PGE2; rodent inflammatory/cancer pain models; inflammatory tumor-bone niche	cAMP/PKA-dependent Na_v_ phosphorylation lowers firing thresholds.
Na_v_1.8/Na_v_1.9	Inflammatory phosphorylation and depolarization; rodent DRG/CIBP models	Sustains repetitive firing and ectopic activity.

#### 2.3.2 Tumor-induced neural remodeling

Beyond direct nociceptor activation, tumors can induce profound structural remodeling of the nervous system. Classical studies utilizing mouse models of breast and prostate cancer bone metastasis have demonstrated that, during tumor invasion and bone remodeling, intraosseous and periosteal sensory nerve fibers (CGRP^+^/TrkA^+^/GAP43^+^) exhibit significant pathological sprouting and reorganization, culminating in tangled, neuroma-like structures (8, 9). Anti-NGF treatment reduces tumor-induced nerve sprouting and neuroma formation together with cancer-related pain behaviors, supporting a functional association between aberrant sensory innervation and pain (10). These structural changes may provide an anatomical substrate for movement-evoked and breakthrough pain, particularly when mechanical loading deforms the periosteum or structurally weakened bone.

However, the precise quantification of tumor-induced neural remodeling becomes increasingly challenging in advanced osteolytic disease. Extensive loss of trabecular and cortical architecture, replacement of the bone marrow by tumor tissue, and disruption of periosteal boundaries may obscure the anatomical landmarks required to define comparable regions of interest and normalize nerve-fiber density. Moreover, neuroma-like sprouts are spatially heterogeneous and three-dimensional, whereas conventional immunohistochemical analyses are generally based on selected two-dimensional tissue sections. Consequently, focal nerve sprouting may be under-sampled, while the destruction or displacement of nerve-bearing tissue at advanced stages may produce an apparent reduction in nerve-fiber density that does not necessarily indicate a true reversal of neural remodeling. Late-stage measurements should therefore be interpreted in relation to disease stage and the extent of structural bone damage and, where feasible, integrated with standardized histomorphometry, serial-section analysis, micro-CT, or three-dimensional imaging approaches (8, 9, 36).

Furthermore, perineural invasion (PNI)—defined as the close association of tumor cells with peripheral nerves or their invasion along or into neural structures—is frequently observed in malignancies such as prostate, pancreatic, colorectal, and breast cancers (37). In bone-metastasis-related contexts, PNI has been linked to a greater probability of subsequent bone metastasis in prostate cancer biopsy cohorts (38). In addition, analysis of paired primary and bone-metastatic specimens from patients with hepatocellular carcinoma demonstrated increased PGP9.5-positive neural infiltration in bone metastases; the authors proposed PNI as a potential contributor to skeletal metastatic progression (39). Overall, current evidence supports a bidirectional but asymmetrically established relationship. Tumor- and bone-derived signals are relatively well supported as drivers of neural sensitization and remodeling in CIBP, whereas evidence that remodeled nerves subsequently drive tumor progression is stronger in selected experimental cancer models and remains less directly established in human bone metastasis and CIBP.

#### 2.3.3 Neurotrophic and axon-guidance signaling driving cancer-associated neurogenesis

A growing body of evidence suggests that tumor cells and their associated stromal cells secrete various neurotrophic factors and axon-guidance molecules—such as NGF, BDNF, GDNF, neuregulins, diverse chemokines, netrins, ephrins, and semaphorins—which promote the migration, outgrowth, and increased branching of nerve fibers toward the tumor microenvironment (TME), leading to “cancer-associated neurogenesis.” Targeting selected neurotrophic pathways, particularly NGF–TrkA signaling, has been shown to suppress tumor-induced neural remodeling and attenuate cancer pain in experimental models. For instance, upon binding to its high-affinity receptor TrkA, NGF not only modulates gene expression in dorsal root ganglion (DRG) neurons via retrograde axonal transport but also induces a dramatic increase in peripheral terminal branching by activating localized signaling pathways. Interventions using anti-NGF antibodies have been shown to inhibit tumor-induced neural remodeling and reduce pain, supporting a functional role for NGF signaling in tumor-induced sensory nerve sprouting and pain (10, 40). Similarly, BDNF contributes to neural plasticity and nociceptive sensitization (41); accordingly, anti-BDNF treatment has been shown to attenuate pain behaviors in experimental bone cancer pain, although direct evidence that BDNF blockade arrests tumor-induced structural neural remodeling remains limited (42, 43).

Beyond these classical neurotrophic pathways, axon-guidance molecules may also couple pathological nerve growth to nociceptive sensitization. In a rat model of bone cancer pain, activation of netrin-1/DCC signaling increased CGRP-positive nociceptive innervation in metastatic bone lesions through downstream FAK/Rac1/Cdc42 signaling, whereas DCC knockdown reduced nociceptive nerve-fiber innervation and ameliorated pain behaviors (44). Ephrin-B2/EphB2 signaling has also been shown to act directly on mouse and human DRG neurons and induce MNK–eIF4E-dependent nociceptor plasticity and hyperalgesic priming (45). In addition, semaphorin signaling can regulate sensory axon growth and pain-related neuronal plasticity; for example, Sema3A supplementation alleviated neuropathic pain and altered DRG signaling through the PI3K/Akt/mTOR–eIF2α pathway in a chronic constriction injury model (46). Nevertheless, unlike netrin-1, the specific contributions of ephrin and semaphorin signaling to neural remodeling and pain in the bone tumor microenvironment have not yet been directly established. These pathways should therefore be regarded as emerging candidate targets rather than validated CIBP mechanisms.

In selected bone metastasis models, neural remodeling is characterized by altered sensory and sympathetic innervation, including pathological sprouting and increased nociceptive sensitivity. Such changes may amplify nociceptive signaling and, in some experimental contexts, influence tumor progression and bone microenvironmental homeostasis (7, 32).

## 3 Peripheral neural regulation of tumor and bone microenvironmental processes

The nervous system is not merely a passive recipient of tumor influence; peripheral neural components can contribute to tumor growth, metastasis, and microenvironmental remodeling (7, 47). In bone metastasis models, peripheral neural and neuron-associated stromal or immune components may influence bone remodeling, tumor colonization, and nociceptive signaling (32).

### 3.1 Neural innervation-driven remodeling of the tumor microenvironment

Nerve fibers infiltrating tumor tissue can remodel the tumor microenvironment (TME) by releasing neurotrophic factors, axon-guidance molecules, neurotransmitters, and neuropeptides (12). Adrenergic signaling, commonly mediated by norepinephrine released from sympathetic fibers, and cholinergic signaling, often associated with parasympathetic input, can influence tumor cells, vasculature, stromal cells, and immune cells. However, these pathways should not be assigned uniformly pro- or antitumorigenic roles, because their effects vary according to cancer type, disease stage, receptor subtype, and the responding cellular compartment (12, 48).

In prostate cancer models, sympathetic nerves and stromal β2/β3-adrenergic receptors contribute predominantly to early tumor development, whereas cholinergic signaling through stromal type 1 muscarinic receptors has been associated with later invasion and dissemination (49). Norepinephrine derived from adrenergic nerves can also activate β2-adrenergic signaling in tumor endothelial cells, suppress endothelial oxidative phosphorylation, and induce an angiogenic switch that supports prostate tumor growth (50). These findings illustrate that autonomic effects may change with disease stage and the cell type receiving the neural signal.

Cholinergic signaling is similarly context dependent. In pancreatic ductal adenocarcinoma models, vagotomy or Chrm1 deletion accelerated tumorigenesis, whereas muscarinic signaling reduced cancer stemness and inhibited MAPK/EGFR and PI3K/AKT pathways, supporting a tumor-suppressive role (51). In contrast, vagal cholinergic signaling promoted gastric tumorigenesis through CHRM3-dependent Wnt signaling and stem-cell expansion, while vagotomy, local denervation, or CHRM3 inhibition suppressed tumor development (52). Thus, parasympathetic or cholinergic signaling is not intrinsically tumor suppressive.

Moreover, acetylcholine may be synthesized and released by cancer cells, forming autocrine or paracrine cholinergic loops (53). Therefore, muscarinic receptor activation does not necessarily indicate direct parasympathetic innervation. Autonomic regulation of the TME should instead be interpreted as a cancer-, stage-, receptor-, and cell-type-dependent process. Importantly, most of these observations derive from non-bone tumor models, and their relevance to skeletal metastasis and CIBP remains insufficiently established.

### 3.2 Direct regulation of tumor cells by neurotransmitters and neurotrophic factors

Tumor cells frequently express an array of receptors for neurotrophic factors and neurotransmitters (such as TrkA, TrkB, and p75^NTR^), enabling them to “sense” and respond to neural-derived signals (e.g., NGF, BDNF, and GDNF) (54). Through these receptor-mediated signaling pathways, the nervous system exerts profound effects on various biological facets of tumor cells, including proliferation, survival, migration, invasion, epithelial–mesenchymal transition (EMT), stemness maintenance, and adaptation to hostile environments, such as hypoxia and nutrient deprivation (47, 54). For instance, in colorectal cancer, BDNF/TrkB signaling has been shown to enhance tumor-cell survival, migration, invasion, and anoikis resistance (55)—that is, the ability of detached epithelial-like cancer cells to evade apoptosis after loss of matrix attachment—thereby facilitating their survival and subsequent metastasis after detachment from the primary tissue.

Beyond classical soluble signals, extracellular vesicles, particularly exosomes, provide an additional route for tumor–nerve communication by transferring proteins, lipids, and regulatory RNAs between cells. Tumor-derived exosomes have been shown to promote neurite outgrowth and tumor innervation; in head and neck squamous cell carcinoma models, this axonogenic activity was potentiated by exosome-associated EphrinB1 (56). Moreover, loss of TP53 in oral squamous cell carcinoma altered the microRNA cargo of tumor-derived extracellular vesicles, including reduced miR-34a and increased miR-21- and miR-324-associated signaling, thereby promoting neuritogenesis and sensory-to-adrenergic neuronal reprogramming (57). The resulting adrenergic neural phenotype subsequently supported tumor growth, illustrating how exosome-mediated tumor-to-nerve signaling can initiate a reciprocal tumor–nerve feedback loop. Nevertheless, direct evidence that nerve-derived exosomal cargo acts on tumor cells, particularly in bone tumors or CIBP, remains limited; therefore, exosome- and microRNA-mediated communication should currently be regarded as an emerging rather than fully established mechanism in the bone tumor microenvironment.

### 3.3 Immune-mediated neural remodeling within the tumor microenvironment

Beyond the direct secretion of neurotrophic factors to induce axonal outgrowth, tumor cells orchestrate neural remodeling by modulating the immune landscape. Macrophages, T cells, and mast cells recruited and activated within the tumor microenvironment (TME) release a spectrum of pro-inflammatory and neurotrophic molecules—including TNF-α, IL-1β, IL-6, NGF, BDNF, CCL2, CXCL1/CXCL12, prostaglandins, histamine, tryptase, and colony-stimulating factors—thereby promoting nerve fiber sprouting, elongation, and phenotypic alteration. Tumor-derived extracellular vesicles and microRNAs may also modulate immune-cell polarization, Schwann-cell responses, and neuronal excitability, but direct evidence in CIBP remains limited (13). The broader immunomodulatory potential of extracellular vesicles is supported by non-cancer studies showing that engineered exosome-based delivery systems can reshape macrophage-associated inflammatory responses (58). Nevertheless, whether analogous mechanisms operate within the bone tumor–nerve microenvironment remains unknown. At peripheral terminals, immune-cell-derived cytokines, prostaglandins, chemokines, and neurotrophic factors activate NF-κB, p38 MAPK, ERK, JAK/STAT, and BDNF-TrkB pathways in nociceptors, increasing excitability and lowering activation thresholds (34, 59, 60). More broadly, cross-trait genetic analyses of chronic inflammatory diseases have identified shared immune-related genetic architecture and tissue enrichment across systemic immune organs and peripheral tissues (61). Although these findings were derived from hypothyroidism and rheumatoid arthritis rather than cancer, they illustrate how common immune-genetic programs may contribute to persistent inflammatory states. Whether comparable systemic genetic–immune mechanisms influence the bone tumor microenvironment or CIBP remains unknown. Persistent peripheral input may subsequently contribute to central sensitization, but detailed spinal mechanisms are outside the scope of this review.

Sensory nerves can also influence immune infiltration by remodeling the tumor microenvironment (TME). Evidence suggests that sensory neurons, through neurotrophic or neurotransmitter signaling, regulate the differentiation and functional states of tumor-associated stromal cells (e.g., fibroblasts and endothelial cells) and immune cells (e.g., macrophages) (62). A recent breast-cancer study showed that sensory neurons stimulated dense extracellular-matrix deposition, creating a physical barrier associated with immune exclusion and impaired effector T-cell access (14). This finding is important for cancer neuroscience but has not yet been demonstrated in bone metastases or linked directly to CIBP. Thus, neural regulation of tumor immunity should be considered model dependent.

### 3.4 Nerve-to-cancer mitochondrial transfer: emerging evidence and unresolved relevance to CIBP

Recent studies have expanded the concept of tumor–nerve communication beyond soluble biochemical signaling to include intercellular organelle transfer. Utilizing MitoTRACER, a genetic reporter designed to trace intercellular mitochondrial transfer, Hoover et al. demonstrated the transfer of neuron-derived mitochondria to cancer cells in breast cancer models (11). In nerve–cancer cocultures, this process occurred predominantly through direct cell–cell contact and was associated with tunneling nanotube-like structures. Cancer cells receiving neuronal mitochondria exhibited increased mitochondrial respiration, ATP production, stemness, and resistance to oxidative and shear stress. Fate-mapping experiments further showed that these cells or their progeny were enriched at distant metastatic sites, suggesting enhanced metabolic adaptability and metastatic fitness (11).

**Figure 2:**
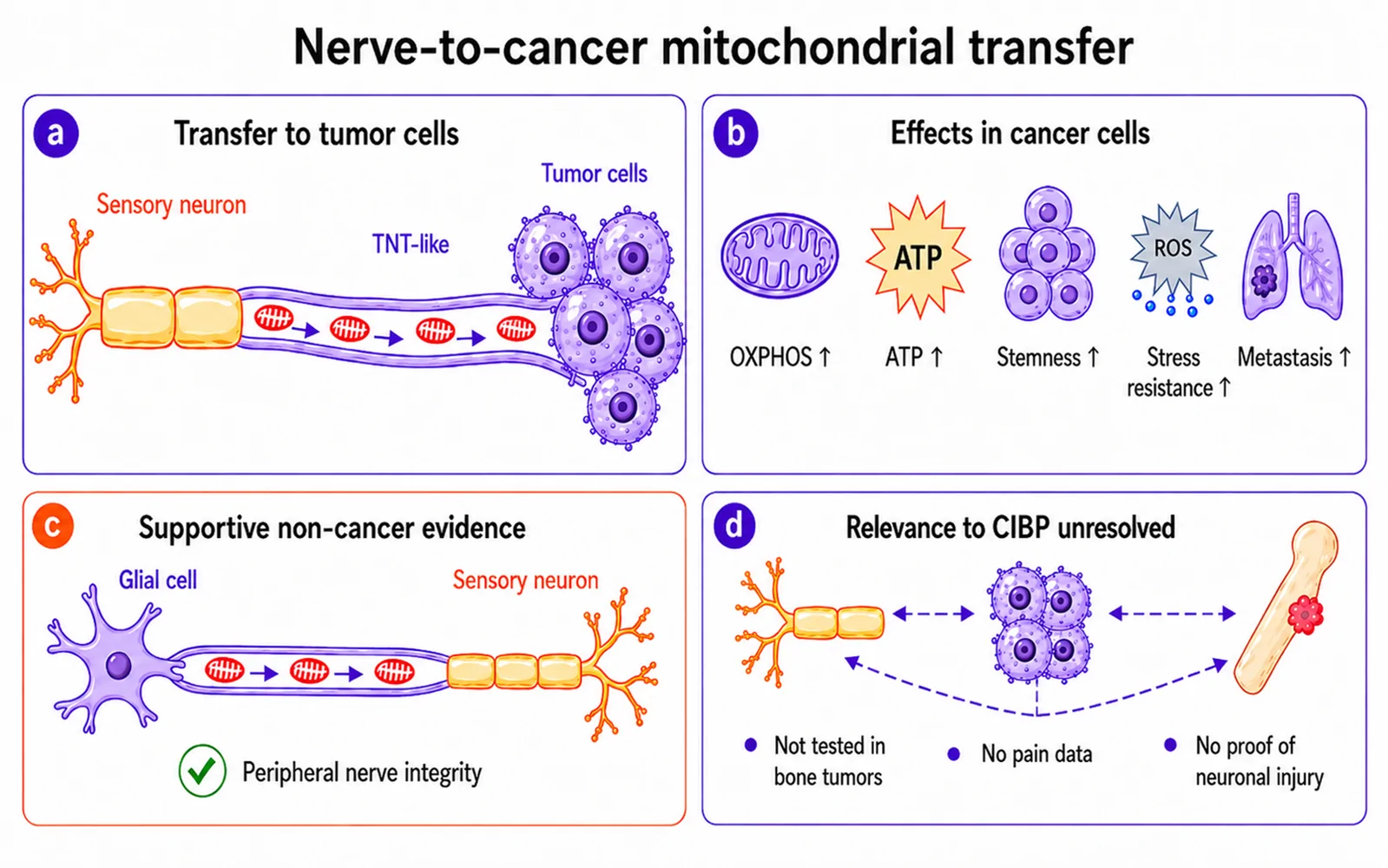
Nerve-to-cancer mitochondrial transfer and its proposed implications. **(a)** Sensory neuron-derived mitochondria are transferred to tumor cells through tunneling nanotube (TNT)-like structures. **(b)** Mitochondrial acquisition by tumor cells is associated with increased oxidative phosphorylation, ATP production, stemness, stress resistance, and metastatic potential. **(c)** Complementary evidence indicates that glial-to-sensory neuron mitochondrial transfer supports peripheral nerve integrity. **(d)** The relevance of nerve-to-cancer mitochondrial transfer to cancer-induced bone pain (CIBP) remains unresolved, with no direct evidence establishing its effects on pain-related phenotypes, neuronal injury, or bone tumor models.

However, this study did not examine bone tumors, skeletal metastases, nociceptor function, neural injury, or pain behavior. Complementary evidence supporting the neurological significance of mitochondrial trafficking has recently emerged from the pain field. Xu et al. reported that satellite glial cells maintain sensory neuron function through TNT-mediated mitochondrial transfer, whereas disruption of this physiological mitochondrial supply led to small-fiber degeneration and neuropathic pain phenotypes (63). These findings demonstrate the importance of mitochondrial exchange for peripheral sensory-neuron homeostasis but do not establish that nerve-to-cancer mitochondrial transfer causes neural injury or CIBP. Therefore, the relevance of this process to the bone tumor–nerve interface and cancer-induced bone pain remains to be directly investigated (**Figure 2**).

### 3.5 Preclinical applications and therapeutic implications

In the experimental models reported by Hoover et al., BoNT/A-mediated chemical denervation reduced nerve-derived mitochondrial transfer or mitochondrial load in cancer cells, whereas lineage-tracing experiments showed that cancer cells acquiring neuronal mitochondria were selectively enriched at metastatic sites (11). These findings support further investigation of nerve-to-cancer organelle transfer as an anticancer target, but they do not establish analgesic efficacy or safety in CIBP (11). Because tunneling nanotubes are F-actin-based structures involved in both physiological and pathological intercellular communication, broadly targeting actin dynamics would be expected to lack TNT specificity and may disrupt normal cellular communication (64, 65). Accordingly, strategies targeting mitochondrial transfer or TNT-like structures remain preclinical and should be evaluated separately for tumor control, neural injury, and pain outcomes.

## 4 Neuron-like phenotypic features in tumor-associated macrophages: preliminary evidence and unresolved questions

**Figure 3:**
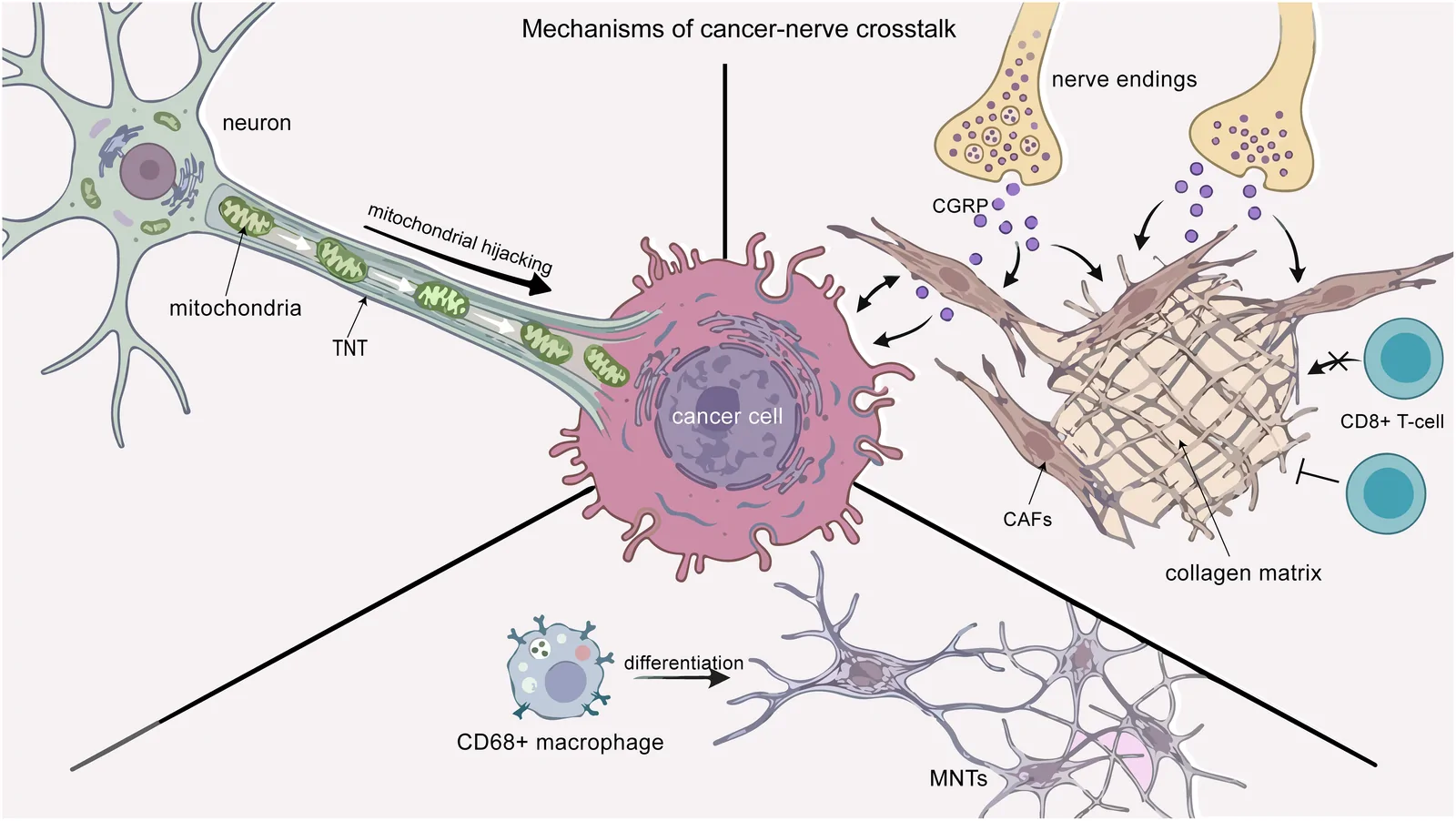
Schematic representation of three emerging and experimentally distinct mechanisms of cancer–nerve crosstalk. Left: cancer cells acquire neuron-derived mitochondria through tunneling nanotubes (TNTs). Upper right: sensory neuron-derived calcitonin gene-related peptide (CGRP) activates cancer-associated fibroblasts (CAFs), promoting collagen deposition and restricting CD8^+^ T-cell infiltration. Lower: CD68^+^ tumor-associated macrophages acquire neuron-like phenotypic features, referred to as macrophage-to-neuron-like transition (MNT). These mechanisms were identified in distinct experimental models and should not be interpreted as an integrated pathway or as established mechanisms of bone metastasis or CIBP. CAFs, cancer-associated fibroblasts; CGRP, calcitonin gene-related peptide; CIBP, cancer-induced bone pain; MNT, macrophage-to-neuron-like transition; TNTs, tunneling nanotubes.

In non-small cell lung cancer models, Tang et al. identified a tumor-associated macrophage (TAM) subset showing neuron-associated gene expression and neurite-like morphology, which they termed macrophage-to-neuron-like transition (MNT) (66). These cells were associated with intratumoral axonal growth and cancer-related nociceptive behavior, and Smad3 was implicated as a regulator within this experimental system (66). More recently, an independent study reported neuron-like macrophage differentiation in nasopharyngeal carcinoma and implicated the APOE–TREM2 axis in tumor-associated chronic pain (67). Nevertheless, the evidence remains limited to a small number of studies in lung and nasopharyngeal carcinoma models, and neither pathway has been demonstrated in bone metastasis or CIBP. Although neuronal marker expression and neurite-like morphology support phenotypic plasticity, they do not establish complete lineage conversion, electrical excitability, or functional synaptic integration. Accordingly, MNT should currently be regarded as a preliminary, model-specific observation rather than an established biological program or therapeutic target (**Figure 3**). Independent lineage-tracing, electrophysiological, and replication studies are required to clarify its biological and clinical relevance (**Table 3**).

**Table 3:** Reported features of neuron-like tumor-associated macrophages and current evidentiary limitations.

Evidence category	Reported observations	Current limitations
Macrophage identity	Macrophage-associated origin and marker expression within TAM populations	Lineage assignment requires rigorous fate mapping
Neuron-associated features	Selected neuron-associated transcripts/proteins and neurite-like morphology in lung and nasopharyngeal carcinoma models	Marker expression and morphology do not establish complete transdifferentiation or electrical excitability
Functional association	Association with intratumoral innervation and cancer-related nociceptive behavior in the reported models	Evidence remains limited to a small number of studies in lung and nasopharyngeal carcinoma models and has not been validated in bone metastasis or CIBP.

## 5 Current and emerging therapeutic strategies

Current clinical management of painful bone metastases combines disease-directed treatment, radiotherapy, surgery or stabilization when indicated, antiresorptive therapy, and multimodal analgesia (2, 4). Radiotherapy, opioids, bisphosphonates, and denosumab are clinically established, whereas most neural and neuroimmune approaches remain investigational (68), and mitochondrial-transfer and nanodelivery strategies remain preclinical (11, 69). The following section therefore separates established clinical options from mechanistically attractive but unvalidated approaches.

### 5.1 Pharmacological treatments and their limitations

Pharmacotherapy remains a primary component of CIBP management. Standard regimens include non-steroidal anti-inflammatory drugs (NSAIDs), opioids, and adjuvant analgesics selected according to nociceptive and neuropathic features (3, 4, 68). Opioids remain important for moderate-to-severe CIBP, but constipation, sedation, nausea, respiratory depression, endocrine effects, tolerance, and misuse risk can limit long-term treatment. Bisphosphonates and denosumab reduce skeletal complications and may improve pain in selected patients by suppressing osteoclast-mediated remodeling, but they do not address all neural and inflammatory components of CIBP. Radiotherapy provides clinically meaningful pain relief for many patients with localized painful bone metastases, although onset, durability, retreatment requirements, marrow exposure, and fracture risk vary (2, 4, 68). Representative clinical trial evidence for established and emerging interventions in painful bone metastases is summarized in **Table 4**.

**Table 4:** Representative clinical trial evidence for established and emerging interventions in painful bone metastases.

Intervention	Representative clinical evidence	Main pain-related findings	Clinical interpretation and limitations
Palliative radiotherapy	Hartsell et al. (70); randomized phase III trial in patients with painful bone metastases	Single-fraction 8 Gy achieved pain relief comparable to 30 Gy in 10 fractions.	Effective standard treatment; retreatment requirements may differ between schedules.
Zoledronic acid	Weinfurt et al. (71); randomized placebo-controlled study in prostate cancer with bone metastases	Zoledronic acid produced a modest improvement in pain outcomes versus placebo.	Analgesic benefit is limited and accompanies its broader effects on skeletal morbidity.
Denosumab	Cleeland et al. (72); randomized phase III study in breast cancer with bone metastases	Denosumab delayed progression from no/mild pain to moderate/severe pain compared with zoledronic acid.	More evident for delaying pain progression than improving established pain.
Tanezumab	Fallon et al. (73); randomized placebo-controlled trial in cancer pain due to bone metastasis	Tanezumab significantly reduced pain versus placebo at week 8.	Provides clinical proof-of-concept for NGF blockade, but RPOA and long-term safety remain major concerns.

### 5.2 Neural-targeted therapies: opportunities and challenges

As the mechanisms underlying neural involvement in tumor progression and pain become clearer, neural signaling hubs have emerged as potential therapeutic targets. NGF-TrkA signaling is among the best-supported peripheral neural targets in skeletal pain. Tanezumab showed analgesic activity in a randomized trial involving cancer pain due to bone metastasis (73), but systemic NGF blockade raises major safety concerns, particularly rapidly progressive osteoarthritis (RPOA). This risk is especially relevant in patients with skeletal fragility because profound analgesia may mask protective pain and alter joint loading. The safety signal does not invalidate NGF biology, but it limits prolonged systemic neutralization. Future strategies may require careful patient selection, lower or intermittent exposure, lesion-targeted delivery, or selective inhibition of downstream TrkA signaling. BDNF-TrkB signaling is mechanistically relevant to pain plasticity, but much of the available evidence involves DRG or central circuits, and clinical evidence for BDNF/TrkB-directed treatment in CIBP is absent (42, 59, 60).

The tumor-associated inflammatory response also contributes to CIBP by activating receptors and ion channels on peripheral nerve terminals. Selective TRPV1 blockade attenuated pain-related behavior in a rodent bone cancer model (35), providing causal preclinical evidence. However, thermoregulation, sensory safety, and translation from homogeneous animal models to clinically heterogeneous bone metastases remain important barriers. CGRP-directed approaches are also of interest, but direct clinical evidence in CIBP is lacking.

### 5.3 Rationale for mechanism-based combination therapy

CIBP arises from concurrent tumor growth, pathological bone remodeling, mechanical instability, inflammation, nociceptor sensitization, and, in some patients, peripheral nerve injury (3, 4, 68). Given this mechanistic heterogeneity, a single-target approach may be insufficient to provide durable control across different disease stages (4, 68). Current multimodal management may combine disease-directed treatment with radiotherapy, antiresorptive agents, opioid or non-opioid analgesics, neuropathic-pain adjuvants, and surgical or mechanical stabilization when indicated (2, 4). Conceptually, future regimens could couple bone- or tumor-targeted delivery platforms with interventions directed at NGF, CGRP, TRPV1, or inflammatory cytokines (34, 35, 69, 73, 74); however, most such combinations remain untested in CIBP. Prospective studies should assess pain intensity, movement-evoked pain, opioid consumption, physical function, skeletal events, tumor outcomes, and adverse effects as distinct endpoints.

### 5.4 Future directions and translational boundaries

Ramesh et al. reported that bupivacaine and the CGRP receptor antagonist rimegepant altered sensory neuron-tumor signaling, tumor growth, angiogenesis, innervation, and immune-cell recruitment in preclinical osteosarcoma models (74). This study provides proof-of-concept evidence but does not establish analgesic or antitumor efficacy in patients, bone metastases, or other CIBP contexts. The reported magnitude of tumor-growth inhibition should therefore be interpreted as model specific. Replication, pharmacokinetic evaluation, dose optimization, safety testing, and clinical trials are required before therapeutic conclusions can be drawn. Nanodrug delivery systems may improve tumor targeting, controlled release, and combination treatment in osteosarcoma (69), but their direct relevance to analgesia and CIBP remains unproven. Biodistribution, immune clearance, manufacturing reproducibility, long-term toxicity, and validated pain outcomes remain major translational barriers.

Spatial transcriptomics, single-cell and multi-omic profiling, advanced neural tracing, and three-dimensional imaging may help identify which neural, immune, and stromal populations are functionally relevant at different disease stages. Future studies should prioritize replication across tumor types, direct measurement of pain-related outcomes, comparison with current standards of care, and explicit separation of tumor-control effects from analgesic effects.
